# Variants in the Kallikrein Gene Family and Hypermobile Ehlers-Danlos Syndrome

**DOI:** 10.21203/rs.3.rs-4547888/v1

**Published:** 2024-06-10

**Authors:** Cortney Gensemer, Tyler Beck, Lilong Guo, Taylor Petrucci, Jordan Morningstar, Isabelle Kornblau, Kathryn Byerly, Rachel Biggs, Amy Weintraub, Kelsey Moore, Natalie Koren, Victoria Daylor, Christina Hastings, Emily Oberlies, Ella R. Zientara, Elsie Devey, Sarah Dooley, Kristina Stayer, Roman Fenner, Katherine Singleton, Sofia Luzbetak, Deatra Bear, Rebecca Byrd, Julianna Weninger, Erika Bistran, Gyda Beeson, Joshua Kerns, Molly Griggs, Charlotte Griggs, Madalyn Osterhaus, Emily Fleck, Jillian Schnaudigel, Shaina Butler, Sydney Severance, Wiley Kendall, Joe R Delaney, Daniel P. Judge, Peng Chen, Hai Yao, Jan Guz, Alexander Awgulewitsch, Steven A. Kautz, Rupak Mukherjee, Robert Price, Fraser Henderson, Steven Shapiro, Clair A. Francomano, Jason C Kovacic, Mark Lavallee, Sunil Patel, Takiy-Eddine Berrandou, Susan A. Slaugenhaupt, David Milan, Amy R Kontorovich, Nabila Bouatia-Naji, Russell A. Norris

**Affiliations:** Medical University of South Carolina

**Keywords:** Hypermobile Ehlers Danlos Syndrome, hEDS, Connective tissues

## Abstract

Hypermobile Ehlers-Danlos syndrome (hEDS) is a common heritable connective tissue disorder that lacks a known genetic etiology. To identify genetic contributions to hEDS, whole exome sequencing was performed on families and a cohort of sporadic hEDS patients. A missense variant in *Kallikrein-15* (KLK15 p. Gly226Asp), segregated with disease in two families and genetic burden analyses of 197 sporadic hEDS patients revealed enrichment of variants within the *Kallikrein* gene family. To validate pathogenicity, the variant identified in familial studies was used to generate knock-in mice. Consistent with our clinical cohort, *Klk15*^*G224D/+*^ mice displayed structural and functional connective tissue defects within multiple organ systems. These findings support *Kallikrein* gene variants in the pathogenesis of hEDS and represent an important step towards earlier diagnosis and better clinical outcomes.

The Ehlers-Danlos syndromes (EDS) are a group of 14 heritable connective tissue disorders characterized by generalized joint hypermobility, tissue fragility, and multi-organ involvement.^1–3^ Despite evidence of familial inheritance, the most common form of EDS, hypermobile EDS (hEDS), lacks a genetically validated cause. The impacts of hEDS can include frequent joint dislocations and subluxations, tendon and ligament laxity, skin hyperextensibility, connective tissue fragility, and chronic pain. Additionally, patients experience various systemic manifestations including comorbidities affecting the gastrointestinal tract, cardiovascular system, and immune system.^2, 3^ The combination of variable symptom presentation, limited clinical awareness, and the absence of genetic markers can result in delays of several decades before a diagnosis is made for hEDS patients, leading to worsened health outcomes.^4^

To identify genetic causes for hEDS, a genetic registry was developed at the Medical University of South Carolina (MUSC). Within this registry, a four-generation family was identified that presented with autosomal dominant hEDS. Eleven family members were enrolled for genetic analysis, of whom five met the clinical diagnostic criteria for hEDS and three were coded as probable due to age and clinical history at time of analyses (Figure 1A, **Figure S1**). Whole exome sequencing (WES) of the proband (IV-1) and a second cousin (IV-4) was performed. Following variant filtering (see methods), four rare and potentially damaging variants were shared between the affected individuals (IV-1 and IV-4). PCR amplification and targeted sequencing of all enrolled family members identified only one of these variants with a perfect phenotype-genotype segregation throughout the pedigree (Figure 1A, C). This variant, located in the Kallikrein serine-protease gene *KLK15* (chr19:50825890-C-T), was also found in affected members of a second family (Figure 1B, **Figure S1**). The single nucleotide polymorphism (SNP) results in a missense change (KLK15 p. Gly226Asp), is rare in the population with a minor allele frequency (MAF) of 0.002 (gnomAD v2.1.1), and is predicted to be damaging with CADD and DANN scores of 24 and 0.998, respectively. To determine relevance to connective tissue biology, RT-PCR was performed and confirmed *KLK15* mRNA expression in glandular and connective tissues isolated from human and mouse biopsies (Figure 1D, E).

KLK15 is part of a contiguous cluster with 14 other members of the Kallikrein gene family on chromosome 19q13.33 (Figure 1F). Given the known involvement of Kallikreins in regulating one another through activation cascades, and their shared expression patterns in connective tissues (**Figure S2, S3**), we evaluated the genetic burden of the entire *KLK* family of genes in a larger hEDS cohort. WES was performed on 197 clinically diagnosed, unrelated hEDS patients and filtered for *KLK* variants with MAFs less than 0.01 (<1%) in gnomAD. A total of 76 variants were identified, with 48 being unique in the cohort and 65 patients having at least one rare variant in a KLK gene (32.8%) (**Figure S4**). A gene-based burden test was used to evaluate enrichment of rare variants in individual *KLK* genes and the entire contiguous gene cluster in hEDS patients (see methods).^5^ Significant enrichment for qualifying variants was observed in 11 of the 15 KLK genes with p-value <0.05 as well for the entire *KLK* gene cluster (considered as whole, p = 2.28×10^−14^) (Figure 1F, **Figure S5**); thus supporting a broad role for Kallikrein genes in hEDS.

To provide functional support for the role of Kallikrein in hEDS pathogenesis, CRISPR-Cas9 was used to create knock-in mice with the corresponding familial KLK15 variant (**Figure S6**). The Achilles tendon, which shows high KLK15 expression in both mice (Figure 1D) and humans (**Figure S2, S3**), was chosen for analysis. This selection was further justified by reports of Achilles tendon ruptures in members of family 1, making it a relevant tissue to assess for structural and functional deficits in the mice. Freshly isolated Achilles tendons from *Klk15*^*G224D/+*^ (N=8) and control mice (N=9) were subjected to mechanical testing. Microcomputed tomography (mCT) analyses demonstrated *Klk15*^*G224D/+*^tendons were similar in overall anatomical dimensions compared to controls (**Figure S7, S8**). Stress-strain curves revealed a larger displacement, higher strain, and lower toe modulus in *Klk15*^*G224D/+*^mice, consistent with a more elastic tissue (Figure 2A–E). This extended toe region occurs without significant changes in deformation at endpoints and is consistent with previous studies on ligament injuries.^6, 7^ As these mechanical changes are indicative of a structural collagen deficit, ultrastructural analyses were performed on Achilles tendons by transmission electron microscopy (TEM) (Figure 2F–H). Image analyses and quantification of collagen fibrils was performed on a total of 20 independent regions throughout tendons from five *Klk15*^*+/+*^ (N=3,240 fibrils) and 24 independent regions from six *Klk15*^*G224D/+*^(N=4,191 fibrils) mice. An overall 20% reduction in fibril diameter was observed in mutant mice compared to controls with p<0.0001 (Figure 2G). Parsing data into 10nm increments revealed smaller collagen fibrils in *Klk15*^*G224D/+*^ tendons compared to controls (Figure 2H), which correlated with increased elasticity and reduced mechanical strength in *Klk15*^*G224D/+*^tendons.^8^

As cardiovascular defects are a relatively common finding within the hEDS population, hearts from *Klk15*^*G224D/+*^(N=6) and *Klk15*^*+/+*^ (N=5) mice were analyzed using echocardiography and histopathology. Although overall cardiac function was normal (**Figure S9**), valve dysfunction was observed in 83% (5/6) of the mutant mice, with 80% (4/5) having demonstrable prolapse of the mitral leaflets (Figure 2I). None of the control animals had detectable defects in valve function. Myxomatous degeneration of mitral and aortic valves, as assayed by Movat’s Pentachrome stain, was evident in 75% (3/4) of mutant mice compared to 0% (4/4) of controls (Figure 2J). Given previous reports on hEDS patients having an increased risk for changes in aortic dimensions^9^, we examined whether this was apparent in the *Klk15*^*G224D/+*^hEDS mouse model. Consistent with these prior reports, *Klk15*^*G224D/+*^had slightly enlarged aortic dimensions, trending toward significance (p=0.0532) (Figure 2K, **S10**).

This study provides the first genetic and biological evidence for the involvement of *Kallikrein* gene variants in hEDS. Through WES of two families, we identified the KLK15 p.G226D variant. Supporting this genetic discovery, *in vivo* data demonstrated that the KLK15 variant causes structural and functional effects across multiple organ systems in a murine model, consistent with an hEDS phenotype. In a cohort of 197 hEDS patients, we observed enrichment of Kallikrein variants, with 32.8% of patients harboring at least one KLK variant. While most *Kallikrein* variants identified are exceedingly rare, the familial *KLK15* variant has a slightly higher MAF. Similar studies have identified causative dominant missense variants with MAFs between 0.0001 and 0.005 in complex Mendelian diseases.^10–13^ Factors such as genetic background, environmental exposures, potential under-diagnosis, incomplete penetrance, and reduced expression likely contribute to the genotype-phenotype correlation in hEDS.

Exploring the molecular consequences of Kallikrein variants will not only uncover mechanisms of normal connective tissue development and disease but also shed light on the comorbidities commonly associated with hEDS. Notably, Kallikreins are known to interact with substrates in the extracellular matrix, influencing the connective tissue environment in both homeostasis and disease.^14^ This class of genes also plays roles in blood pressure regulation and immune cell function, potentially contributing to various comorbidities such as postural orthostatic tachycardia syndrome (POTS) and mast cell activation syndrome, which are frequently observed in hEDS patients.^15^ Relatedly, it is curious that there is a tight interaction between Kallikrein’s and in the innate immune system, specifically the complement system. Kallikreins can cleave complement 3 (C3) and 5 (C5) directly, leading to the generation of C3a and C5a^16^, which are potent anaphylatoxins that enhance inflammation upstream of mast cell activation. Additionally, in certain pathological conditions such as hereditary angioedema (HAE), dysregulation of the interlinked kallikrein system and the complement pathway can lead to excessive inflammation and tissue swelling^17^, features observed in hEDS patients.

Given the autosomal dominant mode of inheritance and the variability of phenotypes associated with hEDS, it is likely that KLK gene variants, such as *KLK15*^*G226D*^, function primarily in a dominant-negative manner. However, given the known synergistic hierarchy of Kallikreins, where they auto-activate and catalyze the activation of downstream Kallikrein enzymes, even subtle changes in expression levels due to loss-of-function alleles may have damaging effects. While we implicate KLK variants in hEDS, they represent just one aspect of the genetic landscape. The absence of damaging Kallikrein variants should not preclude patients from receiving a clinical diagnosis of hEDS. Although our study indicates the likelihood of additional families and sporadic individuals harboring rare monogenic causes, genome-wide approaches hold promise for revealing the complex genetic architecture of hEDS.

Hypermobile Ehlers-Danlos Syndrome is a connective tissue disorder that impacts many tissues and organ systems. Management of symptoms is challenging and often requires an interdisciplinary team of well-informed clinicians who understand the complexities of hEDS. Studies focusing on the pathophysiology of hEDS are in their infancy, underscoring the need for better clinical understanding. Elucidation of genetic causes and biological pathways involved in hEDS is critical for earlier diagnoses, which can reduce the healthcare burden and improve quality of life. This report provides a first critical step toward mechanistic understanding of disease pathogenesis, paving the way for improved diagnostic tools and enhanced prognoses for patients with hypermobile Ehlers-Danlos Syndrome.

## Methods

### Study Design

Familial genetic studies and exon analyses were performed to determine gene variant enrichment in patients with hEDS. A genetically accurate model of hEDS based on the human KLK15 variant was generated. This model was used for *in vivo* assays to test the effect of the KLK15 variant on connective tissue mechanobiology, ligament laxity, collagen fibril diameter, and functional heart diseases. Power analyses were conducted to determine sample size assuming α = 0.05 with a power of 0.80. A series of tests were conducted to analyze connective tissue defects in two independent study groups, a control group and an experimental group of genetically modified animals. The two animal/sample groups were differentiated by genotype. Animal genotype was de-identified, and researchers were blinded to the animals’ genotypes for purpose of the analyses. Genotyping code was held by one individual not associated with measurement calculations. Analyses were conducted by at least two independent investigators who were blinded to genotype. After procurement of all measurements, the code was broken, and genotype/phenotype correlations were presented. The total numbers of replicates are represented in the figure legends.

### Animal studies

All animal experiments were performed under protocols approved by the Institutional Animal Care and Use Committees at the Medical University of South Carolina. Prior to tissue biopsy, mice were euthanized by isoflurane (Piramal) induction, followed by cervical dislocation in line with the Guide for the Care and Use of Laboratory Animals (NIH publication no. 85–23, revised 1996). Combined data for both sexes are shown. All animal experiments were performed in accordance with IACUC procedures and approved protocol number IACUC-2020–00956.

### Human subjects

Studies involving human research were approved by the Institutional Review Board of Partners Healthcare, (Boston, MA) and MUSC (Charleston, SC) and participants provided written informed consent.

### Whole Exome Sequencing

Whole exome sequencing for the proband and her second cousin in Family 1 was performed through the Broad Institute. An aliquot of genomic DNA (100–150ng in 50μL) was used as the input for DNA fragmentation. Shearing was performed acoustically using a Covaris focused-ultrasonicator, targeting 385bp fragments. Following fragmentation, additional size selection was performed using a SPRI cleanup. Library preparation was performed using a commercially available kit provided by KAPA Biosystems (KAPA Hyper Prep with Library Amplification Primer Mix, product KK8504), and with palindromic forked adapters using unique 8-base index sequences embedded within the adapter (purchased from IDT). The libraries were then amplified by 10 cycles of PCR. Enzymatic clean-ups were performed using Beckman Coulter AMPure XP beads with elution volumes reduced to 30μL to maximize library concentration. Following library construction, library quantification was performed using the Invitrogen Quant-It broad range dsDNA quantification assay kit (Thermo Scientific Catalog: Q33130) with a 1:200 PicoGreen dilution. Following quantification, each library was normalized to a concentration of 25 ng/μL, using a 10 mM Tris HCl pH 8.0 solution. All steps performed during the library construction process and library quantification process were performed on the Agilent Bravo liquid handling system.

After library construction, hybridization and capture were performed using the relevant components of IDT’s XGen hybridization and wash kit and following the manufacturer’s suggested protocol, with several exceptions. A single pre-hybridization pool was created. The pre-hybridization pool comprised of 96 unique libraries was created by equivolume pooling of the normalized libraries as well as 5 μL of Human Cot-1 and 2 μl of IDT XGen blocking oligos. The pre-hybridization pool underwent lyophilization using the Biotage SPE-DRY. Post lyophilization, 4 μL of custom exome bait (TWIST Biosciences) along with 13 μL of hybridization mastermix was added to the lyophilized pool prior to resuspension. An initial incubation was performed at 95°C for 30 seconds, after which time the incubation temperature was lowered to 65°C at which it remained overnight. Library normalization and hybridization setup were performed on a Hamilton Starlet liquid handling platform, while target capture was performed on the Agilent Bravo automated platform.

After post-capture enrichment, library pools were quantified using qPCR (automated assay on the Agilent Bravo), using a kit from KAPA Biosystems with probes specific to the ends of the adapters. Based on qPCR quantification, pools were normalized using a Hamilton Starlet to 2nM, and sequenced using Illumina sequencing technology. The flow cells were then analyzed using RTA v.2.7.3 or later. Each pool of whole exome libraries was sequenced on paired 150 cycle runs with two 8 cycle index reads across the number of lanes needed to meet coverage for all libraries in the pool.

CRAM files aligned to hg38 were transformed to BAM files using SAMtools. BAM files were filtered for duplicates by RmDup and mate pairs fixed using FixMateInformation. These processed BAM les were then input into three independent SNV calling tools: VarScan, LoFreq, and FreeBayes. The intersection of both probands with all three calling tools was used in further analysis (N = 48,720 common variants). To narrow likely causal variants, four filtering criteria were used: (1) Population-level minor allele fractions (MAF) from the 1000 Genomes project, with a cutoff of MAF < 0.01, (2) sequencing allelic fraction in the heterozygous range for both probands (average fraction of between 0.4 and 0.6 aligned reads), (3) SnpEff 4.3 to categorize variant type in relation to genes, and (4) PolyPhen-2 to annotate missense protein-coding variants. Variants in other known EDS genes, such as TNXB, were ruled out and 4 total shared variants were identified that fit these criteria. These variants were: POTEJ V1002M, FRG2C D9N, FRG2C L210M and KLK15 G226D. Sanger sequencing for each of these variants throughout the family was performed using the following primer sets: POTEJ V1002M: 5’ GCTATGTTGCCCTGGACTTC 3 and 5’ ATTTGCGGTGGACAATGGAG 3’; FRG2C D9N: 5’ AGGGACGTATAAAAGGCAGGTC 3’ and 5’ ACTAAGCCATTTCCCATCCCC 3’; FRG2C L210M: 5’ CTCTGGTGAGTCTCTCACATGC 3’ and 5’ TCAGGGTGCTCCCAGCTTAG 3’; KLK15 G226D: 5’ TTCTGTTCCATGTCAGGCGG 3’ and 5’ TTCACTCAACCTGAGACCCC 3’ and BioRad iProof High Fidelity PCR Reagents. PCR cleanup or gel extraction of fragments were generated and sent for sequencing of both strands using the same PCR oligomers. Sequence data was aligned using BLAST and analyzed for phenotype/genotype segregation. One variant: KLK15^G226D^ had a perfect phenotype/genotype transmission through the family and was functionally evaluated further as detailed below.

### KLK Variant Annotation and TRAPD analyses

Whole exome sequencing was performed on 205 patients with a clinical diagnosis of hEDS. Library preparations and sequencing reactions were conducted at Azenta US (South Plainfield, NJ, USA) as follows: Genomic DNA sample were quantified using Qubit 2.0 Fluorometer (ThermoFisher Scientific, Waltham, MA, USA). Enrichment probes were designed against the region of interest and synthesized through Twist Biosciences – Twist Human Comprehensive Panel (South San Francisco, CA, USA). Library preparation was performed according to the manufacturer’s guidelines. Briefly, the genomic DNA was fragmented by acoustic shearing with a Covaris S220 instrument. Fragmented DNAs were cleaned up and end repaired, as well as adenylated at the 3’ends. Adapters were ligated to the DNA fragments, and adapter-ligated DNA fragments were enriched with limited cycle PCR. Adapter-ligated DNA fragments were validated using Agilent TapeStation (Agilent Technologies, Palo Alto, CA, USA), and quantified using Qubit 2.0 Fluorometer. Adapter-ligated DNA fragments were hybridized with biotinylated baits. The hybrid DNAs were captured by streptavidin-coated binding beads. After extensive wash, the captured DNAs were amplified and indexed with Illumina indexing primers. Post-captured DNA libraries were validated using Agilent TapeStation (Agilent, Santa Clara, CA, USA) and quantified using Qubit 2.0 Fluorometer and Real-Time PCR (KAPA Biosystems, Wilmington, MA, USA).

The bioinformatics pipeline from FASTQ files to the variant analysis was performed suing the Franklin analysis platform (Genoox, Tel Aviv, Israel). The analysis was performed as follows: FASTQ files aligned to hg19 were transformed to BAM files using BWA. Duplicate reads were marked and filtered in the process. These processed BAM files were then put into two independent SNV calling tools: GATK haplotype caller, and FreeBayes in addition to a Franklin proprietary CNV caller. After removing 1 sample that failed QC and 6 samples that had pathogenic or likely pathogenic variants consistent with other connective tissue disease, 197 exomes were filtered for genes within the KLK locus. Variants were filtered for rare MAF (less than 0,01, < 1%) using gnomAD allele frequencies and assessed for conservation and pathogenicity through UCSC genome browser and dbNSFP, respectively, to generate the list of variants shown in **Figure S4**.

Previous reports have made claims for the involvement of the Methylenetetrahydrofolate reductase (MTHFR) gene.^18^ We performed genetic-follow up analyses in our WES cohort of 197 individuals and did not observe a significant enrichment of the common MTHFR polymorphisms. Chi-square test for trend revealed no association between hEDS patient genotypes for the C677T (p = 0.9864) or A1298C (p = 0.3156) SNPs when compared to Gnomad v2.1.1 non-Finnish European population. Thus, the high population frequency of these variants and lack of replication in our cohort render these common MTHFR variants as unlikely to cause hEDS. For an accurate synopsis of the impact of these specific MTHFR variants on human physiology, we direct the audience to statements released by the Centers for Disease Control (CDC: https://www.cdc.gov/ncbddd/folicacid/mthfr-gene-and-folic-acid.html) and American College of Medical Genetics (ACMG) recommendations.^19^

Associations between relevant variants located in the Kallikrein-related peptidase (KLK) genes and hEDS (N = 197) were tested using a gene-based burden test implemented in TRAPD (Testing Rare vAriants using Public Data) package.^5^ For the dominant test, for each gene, we calculated the number of individuals carrying at least one qualifying variant. We used non-Finnish European populations (N = 64603) summary statistics from gnomAD v2.1.1 as a control group to evaluate the enrichment of rare KLK genes alleles in patients. Also, as sensitivity analysis, we restricted control population to only samples from individuals who were not selected as a case in a case/control study of common disease (N = 24146). Results were consistent between the two analyses (**Figure S5**). Coverage for the variants in gnomAD v2.1.1 were taken into account and coverage summary for all the variants used in our analyses is shown below. For every variant, the mean and median depth of coverage, as well as the proportion of subjects with specific depth coverage values (over 1, 5, 10, 15, 20, 25, 30, 50, 100) are available from the gnomAD database. These statistics have allowed us to ensure that we only consider variants with adequate coverage for reliable detection.

For exome sequencing

**Table:** 

chrom	pos	mean	median	over_1	over_5	over_10	over_15	over_20	over_25	over_30
19	51322484	61.56	58.00	1.00	1.00	1.00	1.00	0.99	0.96	0.89
19	51325114	34.12	32.00	1.00	1.00	0.99	0.95	0.83	0.69	0.55
19	51330148	35.08	36.00	1.00	0.97	0.85	0.74	0.67	0.62	0.57
19	51330349	67.79	71.00	1.00	1.00	1.00	0.99	0.98	0.95	0.89
19	51331017	66.80	67.00	1.00	1.00	1.00	1.00	1.00	0.99	0.97
19	51331063	58.89	57.00	1.00	1.00	1.00	1.00	0.99	0.98	0.96
19	51359565	69.99	66.00	1.00	1.00	1.00	1.00	1.00	0.98	0.95
19	51361451	75.45	77.00	1.00	1.00	1.00	1.00	1.00	1.00	0.99
19	51361469	74.87	76.00	1.00	1.00	1.00	1.00	1.00	1.00	0.99
19	51361822	81.28	84.00	1.00	1.00	1.00	1.00	1.00	1.00	1.00
19	51361850	64.30	60.00	1.00	1.00	1.00	1.00	1.00	0.99	0.97
19	51379798	77.21	88.00	1.00	1.00	1.00	1.00	1.00	1.00	0.99
19	51379940	75.26	86.00	1.00	1.00	1.00	1.00	1.00	0.99	0.97
19	51380302	57.80	57.00	1.00	1.00	1.00	1.00	0.98	0.94	0.86
19	51381793	63.14	46.00	1.00	1.00	1.00	1.00	0.98	0.94	0.84
19	51410258	97.85	100.00	1.00	1.00	1.00	1.00	1.00	1.00	1.00
19	51411751	97.61	100.00	1.00	1.00	1.00	1.00	1.00	1.00	1.00
19	51451958	82.13	87.00	1.00	1.00	1.00	1.00	1.00	1.00	1.00
19	51452136	75.85	76.00	1.00	1.00	1.00	1.00	1.00	1.00	1.00
19	51452193	88.92	100.00	1.00	1.00	1.00	1.00	1.00	1.00	1.00
19	51453283	69.41	100.00	1.00	1.00	1.00	0.98	0.93	0.85	0.76
19	51462454	76.10	76.00	1.00	1.00	1.00	1.00	1.00	1.00	1.00
19	51471329	87.23	96.00	1.00	1.00	1.00	1.00	1.00	1.00	1.00
19	51483507	65.66	65.00	1.00	1.00	1.00	0.99	0.98	0.94	0.88
19	51483678	78.73	100.00	1.00	1.00	1.00	1.00	1.00	0.99	0.98
19	51485072	36.47	38.00	1.00	0.98	0.90	0.80	0.72	0.66	0.61
19	51485135	44.44	44.00	1.00	1.00	0.99	0.96	0.91	0.84	0.77
19	51503923	47.74	45.00	1.00	0.99	0.95	0.83	0.72	0.66	0.63
19	51518714	87.03	99.00	1.00	1.00	1.00	1.00	1.00	1.00	1.00
19	51518761	88.89	100.00	1.00	1.00	1.00	1.00	1.00	1.00	1.00
19	51519144	19.50	14.00	1.00	0.95	0.73	0.50	0.36	0.28	0.23
19	51519354	30.46	31.00	1.00	0.95	0.74	0.62	0.59	0.56	0.52
19	51526473	51.15	54.00	1.00	1.00	0.99	0.96	0.88	0.79	0.70
19	51535330	63.58	100.00	1.00	0.99	0.94	0.82	0.69	0.61	0.58
19	51537321	61.61	100.00	1.00	0.97	0.79	0.63	0.59	0.59	0.58
19	51559851	65.44	51.00	1.00	1.00	1.00	1.00	0.99	0.97	0.91
19	51559963	77.56	84.00	1.00	1.00	1.00	1.00	1.00	1.00	0.99
19	51560037	44.44	38.00	1.00	1.00	1.00	0.98	0.93	0.83	0.71
19	51563174	45.63	35.00	1.00	1.00	0.99	0.93	0.82	0.68	0.57
19	51563300	66.59	65.00	1.00	1.00	1.00	0.99	0.99	0.97	0.93
19	51568272	17.03	15.00	0.99	0.77	0.59	0.51	0.40	0.28	0.19
19	51581350	64.48	63.00	1.00	1.00	1.00	1.00	0.99	0.99	0.98
19	51581412	37.82	36.00	1.00	1.00	1.00	0.99	0.94	0.84	0.70
19	51582098	63.53	49.00	1.00	1.00	1.00	1.00	0.98	0.93	0.84
19	51582808	70.67	89.00	1.00	1.00	1.00	0.99	0.97	0.93	0.87
19	51582895	57.93	72.00	1.00	1.00	0.94	0.80	0.68	0.62	0.60
19	51584924	17.08	16.00	0.98	0.72	0.59	0.53	0.42	0.28	0.16

For genome sequencing

**Table:** 

chrom	pos	mean	median	over_1	over_5	over_10	over_15	over_20	over_25	over_30
19	51322484	33.63	33.00	1.00	1.00	1.00	0.99	0.95	0.82	0.64
19	51325114	31.63	31.00	1.00	1.00	1.00	0.98	0.92	0.78	0.57
19	51330148	31.56	31.00	1.00	1.00	1.00	0.99	0.92	0.79	0.56
19	51330349	32.13	31.00	1.00	1.00	1.00	0.99	0.95	0.80	0.59
19	51331017	31.57	31.00	1.00	1.00	0.99	0.98	0.92	0.78	0.58
19	51331063	32.03	32.00	1.00	1.00	0.99	0.97	0.92	0.79	0.59
19	51359565	31.88	31.00	1.00	1.00	1.00	0.99	0.92	0.77	0.54
19	51361451	32.80	32.00	1.00	1.00	1.00	0.99	0.94	0.82	0.62
19	51361469	32.85	32.00	1.00	1.00	1.00	0.99	0.94	0.82	0.62
19	51361822	32.45	31.00	1.00	1.00	1.00	0.99	0.94	0.81	0.59
19	51361850	32.12	31.00	1.00	1.00	1.00	0.99	0.93	0.79	0.58
19	51379798	32.02	31.00	1.00	1.00	1.00	0.99	0.93	0.79	0.57
19	51379940	32.38	31.00	1.00	1.00	1.00	0.99	0.94	0.78	0.58
19	51380302	31.03	30.00	1.00	1.00	1.00	0.98	0.91	0.74	0.52
19	51381793	30.99	30.00	1.00	1.00	1.00	0.98	0.92	0.78	0.53
19	51410258	31.75	31.00	1.00	1.00	1.00	0.99	0.93	0.78	0.56
19	51411751	32.19	31.00	1.00	1.00	1.00	0.99	0.93	0.80	0.57
19	51451958	32.16	31.00	1.00	1.00	1.00	0.99	0.94	0.81	0.59
19	51452136	29.67	28.00	1.00	1.00	1.00	0.98	0.90	0.71	0.46
19	51452193	29.77	29.00	1.00	1.00	1.00	0.98	0.90	0.71	0.46
19	51453283	32.79	32.00	1.00	1.00	1.00	0.99	0.93	0.81	0.60
19	51462454	29.74	29.00	1.00	1.00	1.00	0.98	0.89	0.72	0.48
19	51471329	29.73	29.00	1.00	1.00	1.00	0.98	0.87	0.69	0.47
19	51483507	30.21	29.00	1.00	1.00	1.00	0.98	0.91	0.72	0.48
19	51483678	30.50	30.00	1.00	1.00	0.99	0.98	0.92	0.74	0.52
19	51485072	31.89	31.00	1.00	1.00	1.00	0.99	0.93	0.78	0.59
19	51485135	31.88	31.00	1.00	1.00	0.99	0.98	0.92	0.77	0.57
19	51503923	30.52	29.00	1.00	1.00	1.00	0.98	0.89	0.74	0.50
19	51518714	30.24	29.00	1.00	1.00	1.00	0.98	0.90	0.72	0.49
19	51518761	30.02	29.00	1.00	1.00	0.99	0.98	0.89	0.72	0.47
19	51519144	33.57	33.00	1.00	1.00	1.00	0.99	0.94	0.84	0.65
19	51519354	32.90	32.00	1.00	1.00	1.00	0.99	0.94	0.82	0.62
19	51526473	28.59	28.00	1.00	1.00	1.00	0.97	0.86	0.68	0.43
19	51535330	31.03	30.00	1.00	1.00	1.00	0.98	0.91	0.76	0.54
19	51537321	31.72	31.00	1.00	1.00	1.00	0.99	0.94	0.79	0.57
19	51559851	32.03	32.00	1.00	1.00	1.00	0.99	0.93	0.80	0.59
19	51559963	32.58	32.00	1.00	1.00	1.00	0.99	0.94	0.82	0.62
19	51560037	30.85	30.00	1.00	1.00	1.00	0.98	0.92	0.74	0.53
19	51563174	27.74	27.00	1.00	1.00	1.00	0.97	0.84	0.64	0.37
19	51563300	29.45	29.00	1.00	1.00	1.00	0.97	0.89	0.73	0.48
19	51568272	27.79	27.00	1.00	1.00	1.00	0.96	0.83	0.63	0.38
19	51581350	31.93	31.00	1.00	1.00	0.99	0.97	0.91	0.76	0.57
19	51581412	31.97	31.00	1.00	1.00	0.99	0.97	0.91	0.78	0.57
19	51582098	33.15	32.00	1.00	1.00	1.00	0.99	0.94	0.81	0.62
19	51582808	32.15	31.00	1.00	1.00	1.00	0.99	0.93	0.78	0.58
19	51582895	30.14	29.00	1.00	1.00	1.00	0.98	0.90	0.72	0.48
19	51584924	32.77	32.00	1.00	1.00	1.00	0.99	0.93	0.80	0.60

In our dataset, the mean coverage depth for the variants of interest from exome sequencing ranges from approximately 17X to 98X, and for genomes sequencing, ranges from 27.7X to 33.6X. The median coverage depth varies from 14X to 100X in exomes and from 27X to 33X in genomes sequencing. In terms of subjects’ coverage, for exomes sequencing, over 58.8% of subjects have coverage over 10X for all variants, and this value reaches 100% for many of the variants. The same is true for coverage over 15X and 20X, with the minimum proportion of subjects with such coverage being 49.9% and 35.7% respectively, and in many cases, this value reaches 100%. For genomes sequencing, over 99.1% of subjects have coverage over 10X for all variants, and this value reaches 100% for many of the variants. The same is true for coverage over 15X and 20X, with the minimum proportion of subjects with such coverage being 96.0% and 82.9% respectively, and in many cases, this value reaches 99.3% and 95.1%. These coverage statistics, both from exomes and genomes sequencing, ensure a high confidence in variant calling, and underline the robustness of our analysis. While it is true that different variants may have different coverage in gnomAD, our analysis takes this into account by considering only those variants that meet our stringent coverage criteria. This ensures that our findings are based on robust variant calling and accurate estimation of the frequencies of these rare alleles. We used two-sided Fisher’s exact test to estimate the enrichment P-values, consistent with previous reports.^5^

### Bgee Expression

To determine if *KLK* genes are expressed in tissues affected in hEDS patients, data mining in the Bgee portal (https://bgee.org/gene/ENSG00000174562/) was initially performed.^20^ Single cell and bulk RNA sequence analyses of connective tissues are poorly represented in most publicly available datasets. Bgee is a database compiled of RNA-Sqa, Affymetrix, in situ hybridization and EST data integrated for comparison of normal gene expression in various wild-type tissues. Bgee curates and integrates these large datasets, including GTEx, with many smaller ones. mRNA expression revealed broad overlap of the KLK genes in various organ systems including connective tissue (**Figure S2**). A deeper analysis of *KLK15* revealed widespread expression with highest levels of mRNA observed in human tissues such as tendons, skin, veins, skeletal muscle, and the gastrointestinal tract (**Figure S3**). Expression scores for tissues with reported expression were retrieved for the entire KLK gene family in humans.

### RT-PCR

Messenger RNA was purified from freshly dissected mouse tissues, frozen human ACL (Articular Engineering CDD-H-6800-F) and human dermal fibroblasts according to RNeasy Kit (Qiagen, 74104) and cDNA synthesis was performed using qScript XLT One-Step RT-PCR Kit (QuantaBiom 76047–074). Primers for mouse Klk15 were as follows: 5’-TGGCGACAAGGTGCTAGAAG-3’, 5’-CGGGCAGGTTTGAAAAGTCG-3’. Primers for human KLK15 were as follows: 5’-AGT TGC TGG AAG GTG ACG AG-3’, 5’-TGG TTT CCC TGA TCC ACT CC-3’. Negative controls included the RT-PCR reactions in the absence of reverse transcriptase. Results were confirmed by Sanger sequencing.

### Generation of Klk15^G224D^ mouse using CRISPR-Cas9

To model the polymorphism identified from our genetic studies, CRISPR-mediated genome editing technology in mouse embryos was utilized to generate a p. Gly224Asp (G224D) substitution in the homologous region of mouse *Klk15* (NP_777354.1). The mouse KLK15 protein has two fewer amino acids than the human ortholog, making the human G226D variant equivalent to the G224D variant in the mouse. Substitution was achieved through a c.671G > A single nucleotide exchange in *Klk15* (NM_174865.1) of zygotes from C57BL/6J mice. Concomitantly, this nucleotide substitution resulted in the creation of a novel Tth111I restriction site suitable for PCR-based genotyping. Synthetic guide RNA (sgRNA; atcacaggggacatcgccccngg) and single-stranded oligonucleotide (ssODN;5’caagtagctgcagacttttgtgtagacgccaggcttggtggtagtatcacaggggacatcgtcccaggagacaatgccctgcagggcacccccacagaccaagggtcctccggagtcaccctg; opposite strand showing single nucleotide exchange in bold and underlined) were procured from Integrated DNA Technologies, Inc. (IDT), which were designed and validated at the Genome Engineering and iPSC Center (GEiC), Washington University in St. Louis, St. Louis, MO; for design strategy and sequences, see **Figure S6**. CRISPR reagents were delivered by electroporation (EP) to single-cell embryos using 1 mm cuvettes and a Gene Pulser Xcell Eucaryotic system (Bio-Rad Laboratories, Inc.); the EP cocktails contained the following concentrations of reagents in Opti-MEM medium (Gibco/Fisher ThermoScientific): 4 μM Klk15 sgRNA, 4 μM Alt-R HiFi Cas9 Nuclease V3 (IDT),10 μM Klk15 ssODN. Electroporation conditions were as follows: square wave with 2 pulses of 30 V for 3 ms separated by 100 ms. Sequence analysis of 44 pups revealed four mice heterozygous for the G224D variant with a deletion or frameshift on the other allele, nine additional frameshift mice, and eight mice with deletions. The 4 founder *Klk15*^*G224D/fs*^ mice were bred to a second generation for true heterozygous (*Klk15*^*G224D/+*^) pups that were used for breeding and the studies detailed below. Genotypes of the CRISPR-Cas9 *Klk15* knock in mice were confirmed using the following primers and Tth111I restriction enzyme (New England Biolabs): 5’-CCCAGATCCACTCCAAGTAGC-3’ and 5’-TGAGTTCTCTTGGGTTGTGTTG-3’. All founder mice and those from F1 generations were genotyped and Sanger sequenced to confirm successful genome editing and germline transmission of the variant (**Figure S6C**).

### Mechanical Testing

**Figure 2:**
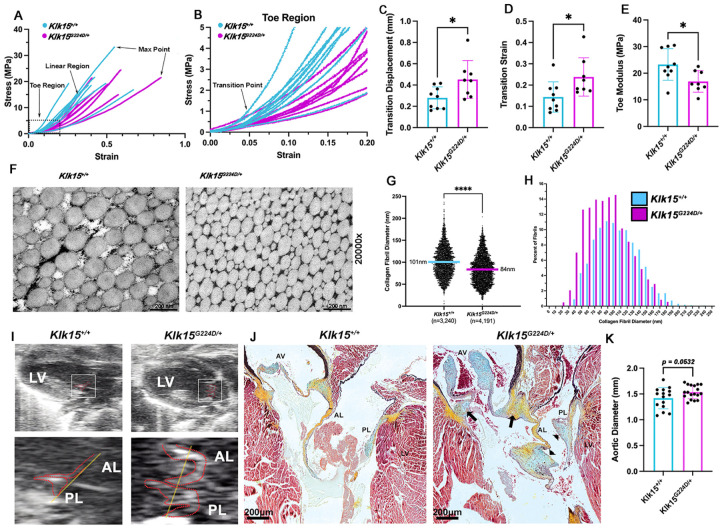
Pathogenicity of the familial KLK15 variant. (**A**) Stress-Strain curves from tensile testing of *Klk15*^*+/+*^ (n=9) and *Klk15*^*G224D/+*^ (n=8) tendons with a focus on the toe region (**B**). (**C-E**) Statistical analysis of transition point displacement, transition strain and toe modulus from tensile testing curves. *<0.05. (**F**) Representative TEMs of Achilles tendon from *Klk15*^*+/+*^ and *Klk15*^*G224D/+*^ mice showing smaller collagen fibrils in the mutant tendons. (**G**) Quantification of fibril diameters from wildtype (n=3,240) and mutant (n=4,191) tendons showing a significant decrease in average fibril diameter in *Klk15*^*G224D/+*^ (84nm vs. 101nm). (**H**) Distribution of fibril diameters in 10 nm increments showing a leftward shift in diameter. ****p<0.0001. (**I**) Echocardiography of 4-month-old wildtype (*Klk15*^*+/+*^;N=5) and mutant mice (*Klk15*^*G224D/+*^; N=6) showing mitral valve prolapse in 5/6 *Klk15*^*G224D/+*^ mice above the level of the annulus (yellow line) (**J**) Movats pentachrome stains revealed myxomatous mitral (arrow heads) and aortic leaflets (AV). Areas of chondrodysplasia and proteoglycan accumulation are evident in the hinge region of the aortic valve (arrows). Red= myocytes, blue= proteoglycans, black=elastin. (**K**) Diameter measurements of *Klk15*^*+/+*^ (N=15) and *Klk15*^*G224D/+*^ (N=18) aorta’s show a trend toward sign cant enlargement in mutant mice (p=0.0532)

Gastrocnemius muscle-Achilles tendon-calcaneus bone samples were first carefully dissected out from the mouse hind leg. Excess soft tissues surrounding the tendon were carefully cleaned off. Each sample was then scanned with microcomputed tomography (μCT) to determine the tendon cross-sectional area^8^, followed with a uniaxial tensile test to obtain the tendon mechanical properties.^6–8^ Samples were stored in a moisture chamber during the sample handling. To determine the tendon cross-sectional area, gastrocnemius muscle-Achilles tendon-calcaneus bone samples were scanned with a Scanco μCT40 system (Scanco Medical, Wayne, PA, USA) at an energy setting of 55 kVP, 145 μA and an isotropic voxel resolution of 18 μm. μCT imaging of each sample was finished within 20 minutes. Tendon region in the cross-sectional image of each sample was segmented and quantified using ImageJ (version 1.52p, National Institutes of Health, Bethesda, MD). After μCT imaging, samples were immediately processed for the mechanical test. The gastrocnemius muscle fibers were carefully scraped off, leaving the tendon fibers intact. Both ends of each sample were gently glued on a thin sandpaper and gripped by custom tensile clamps attached to a mechanical testing system (Bose ELF3200, Bose, MN) with a 5 lbs. load cell. A 0.05 N tare load was first applied to each sample prior to the test. The sample initial length was determined by the clamp-clamp distance at the tare load. Samples were then preconditioned for 8 cycles between 0–5% strain followed with a ramp to failure at 0.01 mm/s. The force and displacement were recorded and analyzed to obtain the mechanical properties (e.g., elastic modulus) at both toe region and linear region using a bilinear fit. The transition point was defined as the point of intersection of the two linear fits of toe regions and linear region. The definition of toe region, linear region, transition point, and maximum point are shown in Fig. 2. Toe modulus is the slope of the fitted line in the toe region while linear modulus (i.e., elastic modulus) is the slope of the fitted line in the linear region. Throughout the mechanical test, samples were kept hydrated with PBS drops. A total of 9 tendons from 5 *KLK15*^*+/+*^ and 8 tendons from 4 *KLK15*^*G224D/+*^ mice were analyzed. Statistical analysis was performed with Mann-Whitney U test using Graphpad Prism.

### Transmission Electron Microscopy

Achilles tendons were freshly dissected from 10-week-old mice and fixed in 2% glutaraldehyde (TAAB, UK) in phosphate buffered saline (PBS) and processed for transmission electron microscopy. Briefly, samples were rinsed in PBS, and post-fixed in 1% osmium tetroxide (Merck, NJ) and 1.5% potassium ferrocyanide in PBS, dehydrated in a graded series of ethanol, and embedded in PolyBed 812 (Polysciences, PA) using acetonitrile as the transitional fluid. Ultrathin sections were stained with 2% uranyl acetate and Hanaichi’s lead citrate (0.15% lead nitrate, 0.15% sodium acetate, 1% sodium citrate dissolved in 41 mL water and 9 mL of 1 N sodium hydroxide (Fisher Scientific) and examined with a JEOL 1400 + electron microscope (JEOL, Japan). Consistent with prior studies on TEM analyses from tendon,^21–24^5 control and 6 mutant mice were used for measurement of collagen fibril diameter and distribution. Micrographs (four per group) from non-overlapping regions of the tendon were taken from cross-sections. Thus, a total of 20 and 24 independent micrographs were analyzed per control and mutant tendons, respectively. Quantification of collagen fibril diameters were measured for each micrograph in entirety using the Photoshop measure tool. A total of 3,240 collagen fibrils from *KLK15*^*+/+*^ and 4,191 collagen fibrils from *KLK15*^*G224D/+*^ mice were analyzed blinded to genotype by two independent investigators. Graphs and statistics were generated using GraphdPad Prism and Mann Whitney U Test. TEM processing and data acquisition were performed in collaboration with the University of South Carolina School of Medicine Instrumentation Resource Facility (IRF).

### Echocardiography

Echocardiographic images of four-to-six-month hearts were acquired in the parasternal long axis and short axis views (40 MHz probe, Visualsonics 3100, Fujifilm, Ontario, Canada) from *Klk15*^*+/+*^ (N = 5) and *Klk15*^*G224D/+*^ (N = 6) mice. End-diastolic frames (coincident with the peak of the QRS complex) and end-systolic frames (smallest volume within a cardiac cycle) were used to determine chamber volumes. M-mode images from the short axis views were used to determine wall thicknesses and LV mass. The diameter of the ascending aorta was measured at the sinotubular junction from ventricular end diastole frames using the “leading edge to leading edge” methodology.^25^ Aortic measurements were averaged over three consecutive, non-respiratory cardiac cycles.

### Movat’s pentachrome staining

Hearts from *Klk15*^*+/+*^ (N = 4) and *Klk15*^*G224D/+*^ mice (N = 4) at 4 months were fixed in zinc formalin (Sigma, Cat No: Z2902), embedded in paraffin, and sectioned at 5 μm. Deparaffinized sections were rehydrated through a graded series of ethanol’s to distilled water. Movat’s pentachrome staining was performed according to manufacturer’s manual (Poly Scientific R &D Corp, Cat No: K042). All samples were cover-slipped using Epredia cytoseal mountant (Epredia, Cat No: 83124). Images of mitral valve sections were captured throughout the regions of the mitral valve leaflets with Keyence BZ-X810 microscope (Keyence, IL). Files were transferred to Adobe Photoshop for labeling and figure preparation.

## Figures and Tables

**Figure 1:**
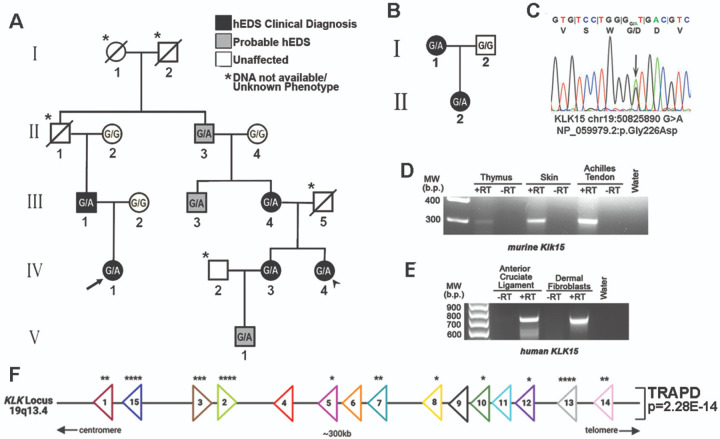
Kallikrein gene variant identification and enrichment in hEDS (**A**) Pedigree of a multigenerational family with autosomal dominant hEDS. Black circles and squares represent those with a clinical hEDS diagnosis. Grey indicates those who are probable-hEDS. Unaffected individuals are black and unknown phenotypes and those whose DNA was not available are marked with an asterisk. Circles and squares represent females and males, respectively. WES was performed on the proband (IV-1) and IV-4 (arrow and arrowhead). (**B**) Pedigree of Family 2 showing genotype of *KLK15*^*G226D/+*^ allele (G/A) in affected I-1 and II-1. (**C**) Chromatogram showing missense KLK15 variant (G/A). (**D, E**) RT-PCR for *Klk15* mRNA in a subset of relevant murine and human connective tissues. RT(+/−) is presence or absence (negative control) of reverse transcriptase enzyme (**F**) *KLK* locus at 19q13.33 shows the proximity and directionality of individual *KLK* genes. Asterisks for each gene represent statistical enrichment in hEDS patients for KLK variants (*<0.05, **<0.01, *** <0.001 ****<0.0001), and the *p-value* of the entire locus (P=2.28E-14).
